# Antioxidant and Anticancer Potentials of Apple Peel and Fruit Extracts: A Combined Docking and Chemical Composition Study

**DOI:** 10.3390/cimb48040343

**Published:** 2026-03-25

**Authors:** Ayla Hançer, Gülşen Güçlü, Ömer Kayır, Serkan Kapancık, Esra Uçar, Burak Tüzün

**Affiliations:** 1 Hotel, Restaurant and Catering Services Department, Gürün Vocational School, Cumhuriyet University, 58140 Sivas, Türkiye; aylahancer@cumhuriyet.edu.tr; 2 Health Programmes Department, Health Services Vocational School, Cumhuriyet University, 58140 Sivas, Türkiye; gulsenguclu@cumhuriyet.edu.tr; 3 Scientific, Technical, Application and Research Center, Hitit University, 19030 Çorum, Türkiye; omerkayir@hitit.edu.tr; 4 Department of Biochemistry, Faculty of Medicine, Sivas Cumhuriyet University, 58140 Sivas, Türkiye; skapancik@cumhuriyet.edu.tr; 5 Plant and Animal Production Department, Technical Sciences Vocational School of Sivas, Sivas Cumhuriyet University, 58140 Sivas, Türkiye; eucar@cumhuriyet.edu.tr

**Keywords:** antioxidant, anticancer, chemical composition, in silico, molecular docking

## Abstract

The apple (*Malus domestica* Borkh.) is one of the most widely consumed fruits worldwide due to its pleasant sensory properties and rich phytochemical composition. Therefore, the present study aimed to comprehensively investigate the chemical composition, antioxidant activity, anticancer effects, and molecular interactions of peel and pulp extracts of the Hünkar apple cultivar collected from different locations, using a combined experimental and computational strategy. These factors had a big effect on the extracts’ phenolic composition and biological activity. Moreover, the anticancer results were corroborated by molecular docking analyses, which offered further understanding of the interactions between bioactive compounds and cancer-associated target proteins. This integrative approach underscores the impact of both biological and methodological variables on the antioxidant and anticancer properties of apple-derived extracts, reinforcing their potential as natural sources of bioactive compounds. Cytotoxic activity against HT-22 and C6 cell lines was evaluated using the MTT assay, showing dose- and time-dependent antiproliferative effects. Apple extracts exhibited anticancer effects that were dependent on dosage and duration. The activities of chemicals found in extracts of Hünkar apple samples collected from four different locations against brain cancer proteins (PDB ID: 2DME, 6YPE, 1RV1) were examined. ADME/T analysis was then performed on the three molecules with the highest activity. The quantum chemical properties of these three molecules were also examined using the Gaussian package program with B3LYP, HF, M062X level in 6–31g, 6–31++g, and 6–31++g(d,p) basis sets.

## 1. Introduction

The apple (*Malus domestica* Borkh.) is among the most widely consumed fruits worldwide, owing to its pleasant aroma and rich nutritional composition. In terms of fresh fruit consumption, apples currently rank fourth globally [1,2]. Apples (*Malus domestica*) belong to the Rosaceae family, and more than 7500 cultivars have been identified worldwide, of which only about 20 are commercially cultivated [3].

Apple production is primarily concentrated in temperate and cold climatic regions, yet cultivation now extends across nearly all continents. Accordingly, the apple represents a crop of strategic importance in global agriculture [4]. According to FAOSTAT data [5], global apple production reached approximately 97.34 million tons in 2023. Nearly half of this production—around 49.6 million tons—was contributed by China, followed by the United States with 5.15 million tons and Türkiye with 4.6 million tons. In Türkiye, the most widely cultivated apple variety is *Starking*, followed by *Golden*, *Amasya*, and *Granny Smith* cultivars [6].

Apples have a very pleasant taste and a balanced composition. In terms of nutritional value, apples are rich in dietary fiber, monosaccharides, minerals, natural antioxidants such as polyphenolic compounds and ascorbic acid (vitamin C) [7,8]. Apples, recognized as a significant source of phytochemicals, have been shown through epidemiological studies to positively impact various lifestyle-related diseases. Among the factors affecting the chemical composition of apples, variety, cultivation techniques and planting year have an important place. However, the properties of chemical compounds may react in different ways to thermal, mechanical and biochemical processes [4,9].

The chemical components of apples have sparked a lot of curiosity, especially because of their potent antioxidant qualities. The primary components of apples’ antioxidant capacity are phenolic compounds, which are one of the natural antioxidant groups [10]. Apple polyphenols are distributed differently; the peel has the highest percentage of polyphenols, whereas the inner half of the fruit has a much lower percentage [11]. Anthocyanins, hydroxycinnamic acids, hydroxybenzoic acids, flavanols, flavonols, and dihydrochalcones are among the many phenolic chemicals found in apples [12]. The ratio of phenolic chemicals is affected by the environment, genetics, variety, and culture techniques [13]. Phenolic chemicals are found in apples, and they are well known for their many health benefits. Patocka et al. [14] and Arnold and Gramza-Michalowska [12] say that these include strong antioxidant properties, anti-inflammatory effects, lowering cholesterol, protecting the heart, fighting diabetes, and fighting cancer.

Research has demonstrated that the bioactive compounds present in apples may significantly contribute to cancer prevention and treatment. Apple peel extracts have demonstrated tumor formation inhibitory effects on breast cancer cells [15], and studies indicate that consuming multiple servings of apples daily may reduce the risk of colorectal cancer [6]. It has also been noted that phenolic extracts from Gala variety apples (*Malus domestica* Borkh cv. Gala) protect cellular DNA from UV radiation and may be utilized in melanoma treatment formulations or cosmetic products [16]. Apples contain a flavonoid called phloretin, which has been shown to fight cancer in people with non-small-cell lung cancer [17], help treat prostate cancer [18], and help treat human cervical cancer cells [19].

Computational chemistry and molecular modeling have become essential for understanding the structural and electronic properties of bioactive molecules in recent years [20]. Theoretical calculations, including quantum chemical analyses and molecular docking studies, facilitate the elucidation of atomic-level molecular interactions, thereby corroborating experimental findings in natural product research [20]. These methods give us useful information about how stable, reactive, and well phytochemicals bind to biological targets. ADME/T (Absorption, Distribution, Metabolism, Excretion, and Toxicity) analyses also help us guess how safe and effective a new drug might be [21]. It is possible to get a full picture of the structure–activity relationships of natural compounds by combining these computational methods with experimental assays [22]. In this context, the theoretical examination of the bioactive constituents of Hünkar apple extracts provides a significant framework for clarifying their anticancer mechanisms and evaluating their potential as lead compounds in drug development.

Anatolia is known as a major source and natural habitat for apples, and it has a lot of different genetic types. There are many local genotypes in the area, which shows how rich its apple genetic resources are [23,24]. Gürün district is in the south of Sivas and has been used as a place to live at different times in history. It is also the meeting point for important transportation networks [25,26]. Sarı Sultan, Şah, and Ayvaniye are some of the local apple varieties that grow in the area. The Hünkâr apple is another important local variety that is grown in Gürün. It is the most common apple variety grown in the district. This apple species, which is usually picked in the middle of October, has a very special place [27]. This study aimed to evaluate the antioxidant and anticancer properties of the flesh and peel of the Hünkâr apple cultivar cultivated in the Gürün district of Sivas. The activities of chemicals found in extracts of Hünkar apple samples collected from four different locations against brain cancer proteins (PDB ID: 2DME, 6YPE, and 1RV1) [28,29,30] were examined. ADME/T analysis was then performed on the three molecules with the highest activity. The quantum chemical properties of these three molecules were also examined using the Gaussian package program with B3LYP, HF, M062X level [31,32,33] in 6-31g, 6-31++g, and 6-31++g(d,p) basis sets.

## 2. Materials and Methods

### 2.1. The Collection of Apple Samples

Apple samples of the “*Hünkar*” cultivar were obtained from local producers in four different regions of Gürün, namely Suçatı (SCT), Burçevi (BRC), Kurultay (KRL), and Şuğul (SGL). The fruits were harvested at commercial maturity and carefully transported to the laboratory. Damaged and bruised parts were removed, and the apples were thoroughly washed and air-dried. Subsequently, the apples were peeled with a stainless-steel knife, quartered to remove the cores, and sliced into pieces approximately 1–3 mm in thickness. The slices were then spread evenly on clean cloths and dried at room temperature under ambient conditions. After drying, the apple samples were pre-cut into smaller pieces using kitchen scissors and further ground for 30 s with a coffee grinder to obtain a fine powder suitable for extraction.

### 2.2. The Preparation of Extracts

The extraction process, methanol, ethanol, and distilled water were used as solvents to prepare the extracts of apple peel and fruit samples for subsequent analyses.

The samples were continuously agitated for 24 h, after which the mixtures were filtered through Whatman No. 1 filter paper. The obtained filtrates were concentrated to dryness under reduced pressure using a rotary evaporator (Heidolph Scientific Products GmbH, Schwabach, Germany) maintained at 40 °C, and this process was repeated three times. All experimental analyses were performed in triplicate at the CÜTAM Laboratory of Sivas Cumhuriyet University, employing a randomized sampling approach.

### 2.3. The Chemical Composition

The analyses were performed using a Thermo Scientific Dionex Ultimate 3000 UHPLC (Thermo Fisher Scientific, Germering, Germany) system coupled to a tandem mass spectrometer (TSQ Quantum Access Max; Thermo Fisher Scientific, San Jose, CA, USA). Data acquisition and processing were carried out using Xcalibur 2.2 software. The ionization source employed was H-ESI (Heated Electrospray Ionization). The mobile phase flow rate was set at 0.7 mL/min, and the column oven temperature was maintained at 30 °C. The injection volume was 20 µL, and separation was achieved using an ODS Hypersil column (4.6 × 250 mm, 5 µm). The total analysis time was 34 min.

### 2.4. The Antioxidant Activity

The antioxidant potential of the extracts was evaluated using several in vitro assays. The DPPH free radical scavenging activity was determined according to the method originally described by Blois [34], with minor modifications. Similarly, the ABTS radical scavenging assay was performed following a modified version of the procedure proposed by Re et al. [35]. The total phenolic content (TPC) was measured spectrophotometrically following the method of Clarke et al. [36], and the results were expressed as milligrams of gallic acid equivalents per gram of dry weight (mg GAE/g). The total flavonoid content (TFC) was determined using the aluminum chloride colorimetric method described by Molan and Mahd [37], and the results were expressed as milligrams of rutin equivalents per gram of dry weight (mg RE/g extract).

### 2.5. The Cytotoxic Activity

#### 2.5.1. The Working Protocol for Cell Culture

HT-22 (Hippocampal neuronal cell line) and C6 (glioma) cell lines were obtained from ATCC. Dulbecco’s Modified Eagle Medium (DMEM) supplemented with 10% Fetal Bovine Serum (FBS) and 1% penicillin–streptomycin was used for cell culture. Cells were grown in an incubator at 37 °C, 95% humidity, and 5% CO_2_.

#### 2.5.2. Determination of Effective Dose Levels for 24, 48 and 72 h in Cytotoxicity Studies Using the MTT Method

Determination of cytotoxic activities in HT-22 and C6 cell lines for 24, 48 and 72 h was carried out by MTT method. Cytotoxic experiments were carried out when the cell density reached 80–90%. For MTT determination, cell suspensions (with a cell density of 1 × 10^5^/mL) in DMEM supplemented with 10% FBS and 1% penicillin–streptomycin were seeded in 96-well plates. Cells exposed to apple extracts at doses ranging from 100 to 1000 µg/mL were incubated for 24, 48 and 72 h. After incubation, 10 µL of MTT was added to each well. After adding MTT, the cells were incubated for 3 h at 37 °C in a 5% CO_2_ incubator. MTT was aspirated from the cells. Then, 100 µL of dimethyl sulfoxide (DMSO) was added to each well and incubated for 15 min. To determine cytotoxic activities, absorbance values were read at 570 nm with a microplate reader. Data was analyzed using the GraphPad Prism 12.0 method. Graphs were generated for the cytotoxic activities of the cells.

#### 2.5.3. Statistical Analysis

Determination of cytotoxic activities in HT-22 and C6 cell lines for 24, 48 and 72 h was performed in 3 replicates. Results are given as mean ± SEM. Study data were analyzed by one-way ANOVA method (* *p* < 0.05, ** *p* < 0.01 and *** *p* < 0.001).

### 2.6. Molecular Docking

Molecular docking approach was adopted to compare the interaction of the plant compounds with biological materials, proteins. Molecular docking simulations were conducted using a Schrödinger’s Maestro Molecular Modeling platform (version 13.4) [38]. It consisted of multiple parts and each of them was executed distinctly. The initial phase involved examining the interactions between the plant compounds and the proteins, by using the Glide ligand docking [39,40], and the LigPrep [41] modules, and the protein preparation module [42]. All computations were conducted using the OPLS4 approach. ADME/T analysis (absorption, distribution, metabolism, excretion, and toxicity) was made to assess the pharmacological potential of the plant compounds. Impacts and responses of the plant compounds in human metabolism were anticipated utilizing the Qik-prop module of the Schrödinger software [43].

In addition to the primary targets, the protein structure with PDB ID: 1RV1 was included in the docking study to provide a broader evaluation of ligand–protein interactions. The selection of 1RV1 was based on its well-characterized three-dimensional structure, high crystallographic resolution, and the presence of a clearly defined active binding pocket, which makes it suitable for reliable docking simulations. Furthermore, this protein has been widely utilized in previous computational studies as a representative biological target for assessing binding affinity and interaction profiles. Therefore, incorporating 1RV1 into the docking protocol allowed for a more comprehensive and comparative analysis of the interaction behavior of the studied compounds.

### 2.7. Gaussian Calculations

A significant amount of information on the chemical and biological characteristics of molecules may be obtained via the use of theoretical calculations. Theoretical computations are used to gather a great deal of information about quantum chemical parameters. To provide an explanation for the chemical activities of the molecules, the parameters that were computed are used. To compute molecules, a wide variety of applications are used. Gaussian09 RevD.01 and GaussView 6.0 are the names of these kinds of apps [44,45]. The 6-31++g(d,p) basis set was used in the computations that were carried out using these programs. The B3LYP, HF, and M06-2x techniques were utilized. Numerous quantum chemical parameters have been discovered as a consequence of these computations for quantum chemistry. There are a variety of chemical properties of molecules that are described by each parameter. Using the following formula, the theoretical parameters are computed [46].

$$\chi =-{\left(\frac{\partial Ε}{\partial Ν}\right)}_{\upsilon \left(r\right)}=\frac{1}{2}\left(I+A\right)≅\frac{1}{2}(E_{HOMO}+E_{LUMO})$$

$$\eta =-{\left(\frac{\partial^{2}Ε}{\partial {Ν}^{2}}\right)}_{\upsilon \left(r\right)}=\frac{1}{2}\left(I-A\right)≅-\frac{1}{2}\left(E_{HOMO}-E_{LUMO}\right)$$

$$\sigma =1/\eta \;\;\omega =\chi^{2}/2\eta \;\;\varepsilon =1/\omega$$

In these equations, χ represents the electronegativity, which reflects the tendency of a molecule to attract electrons, while η denotes the chemical hardness, describing the resistance of a chemical species to charge transfer. The parameter σ corresponds to the chemical softness (σ = 1/η), indicating the reactivity of the molecule. The electrophilicity index (ω = χ^2^/2η) measures the stabilization energy when the system acquires additional electronic charge from the environment, whereas ε represents the inverse of electrophilicity (ε = 1/ω), describing the nucleophilicity tendency. In addition, E_HOMO_ and E_LUMO_ correspond to the energies of the highest occupied molecular orbital and lowest unoccupied molecular orbital, respectively, and I and A represent the ionization potential and electron affinity calculated according to Koopmans’ theorem.

## 3. Results

### 3.1. Molecular Composition Analysis

The LC–MS analysis revealed that the phenolic composition of Hünkar apple extracts varied markedly depending on the solvent type, fruit part, and sampling location. In general, methanolic and ethanolic extracts exhibited higher concentrations of phenolic compounds compared to aqueous extracts, indicating that polar organic solvents were more efficient in extracting phenolics and flavonoids from apple tissues. Hernández-Carranza et al. [47], Reis et al. [48], and Asma et al. [49] reported that while water can effectively extract hydroxycinnamic acids and flavonoids, it is not suitable for quercetin glycosides; they also emphasized that methanol is particularly efficient for extracting chlorogenic acid. Methanol has a stronger ability to dissolve things and a better ability to mix with other things than water, which makes extraction faster and more effective [50].

Additionally, the peel had a lot more phenolic compounds than the fruit pulp. This is in line with what has been found before, which suggests that these bioactive compounds mostly build up in the outer layers of the fruit’s cells. Boyer and Liu [51] showed that the peel of an apple has a lot more phenolic compounds than the pulp.

The highest amounts of chlorogenic acid were found in BRC pulp extracted with ethanol (7829.8 µg/g) and methanol (7057.074 µg/g). SCT pulp extracted with ethanol (6753.302 µg/g) and KRL pulp extracted with ethanol (6533.1 µg/g) were next, confirming that this compound is important in the fruit matrix. Catechin and epicatechin were also present in significant amounts, especially in peel extracts made with ethanol and methanol. For example, SCT peel (EtOH) and KRL peel (MeOH) had catechin levels of 1144.9 µg/g and 889.1 µg/g, respectively. SCT peel (EtOH) had epicatechin levels of up to 3022.1 µg/g in Table 1.

These flavanols are known to help apples fight off free radicals, and the fact that they are more common in peel samples backs up the idea that they are more effective at doing so, as the peel serves as the primary protective barrier against environmental stressors, leading to a higher accumulation of antioxidant compounds in this tissue compared to the pulp.

Caffeic acid was detected in moderate amounts, mainly in aqueous extracts, with the highest value in SCT peel (Water, 537.1 µg/g). Meanwhile, quercetin and rutin, two major flavonols, were primarily found in methanolic and ethanolic peel extracts. The SGL peel (EtOH), SGL peel (MeOH), and SCT peel (EtOH) samples exhibited high levels of quercetin (206.00, 162.45, and 156.58 µg/g, respectively), while rutin reached its highest concentration in KRL peel (EtOH), KRL peel (MeOH), SCT and SGL peel (EtOH) samples (616.334, 562.312, 542.2 and 572.9 µg/g, respectively). The abundance of these compounds in the peel supports the view that this part of the fruit is the richest source of flavonoids and antioxidant constituents. Other researchers have also supported this finding, reporting that some phenolic compounds are more abundant in apple peel than in the flesh. In addition, these researchers reported that apples contain hydroxycinnamic acids such as quinic and caffeic acids, as well as chlorogenic, *p*-coumarylquinic, and *p*-coumaric acids [12,49,52]. Cuthbertson et al. [53] reported that the apple fruit contains polyphenolic metabolites such as (+)-catechin, (−)-epicatechin, phloridzin, quercetin, cyanidin, and chlorogenic acid. Dhyani et al. [54] suggested that apples should be consumed without peeling, as the peel contains a higher concentration of phenolic compounds.

In terms of regional differences, the SGL and SCT samples had the most varied and plentiful phenolic profiles, followed by KRL. The BRC samples, on the other hand, had less varied phenolic profiles. The BRC pulp ethanol extract exhibited a significantly elevated concentration of chlorogenic acid, while other phenolic compounds were present at reduced levels, indicating a varietal and tissue-specific distribution. These results underscore the impact of the cultivation site on the accumulation of secondary metabolites. According to Dhyani et al. [54], the phenolic composition of apples is affected by the altitude at which they are grown.

There are two main groups of phenolic acids: benzoic acid derivatives (like gallic acid) and cinnamic acid derivatives (like coumaric, caffeic, and ferulic acids). Caffeic acid is one of the most common phenolic acids found in fruits and vegetables. It often forms an ester with quinic acid, which is called chlorogenic acid and is the main phenolic compound in coffee [55,56]. This classification aligns with the phenolic composition observed in the current study, which identified cinnamic acid derivatives, including chlorogenic and caffeic acids, as the predominant compounds in apple peel and pulp extracts.

These results show that both biological and methodological factors, such as the extraction solvent, plant tissue, and geographical origin, are very important in deciding the phenolic composition of Hünkar apple extracts. The prevalence of chlorogenic acid, catechin, epicatechin, and quercetin compounds signifies that these phenolics significantly enhance the antioxidant capacity of Hünkar apples. Their elevated concentrations in the peel further underscore its role as a primary source of bioactive phenolic antioxidants.

### 3.2. The Antioxidant Activity Result

#### 3.2.1. ABTS (2,2′-Azinobis(3-ethylbenzothiazoline-6-sulfonic acid)) and DPPH (2,2-Diphenyl-1-picrylhydrazyl)

**Figure 1:**
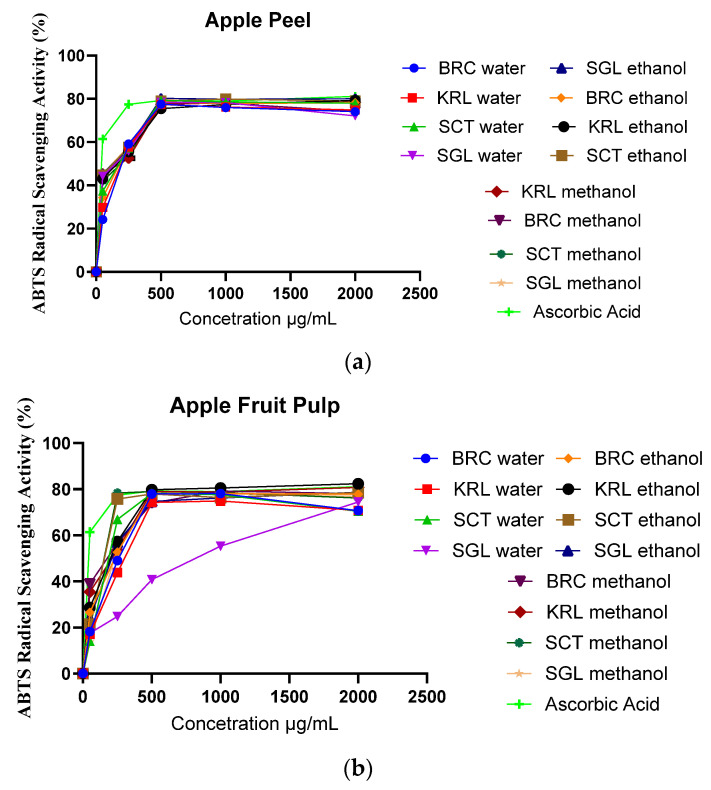
ABTS activity (µg/mL) of apple peel (BRC, KRL, SCT, and SGL) extracts (**a**) and apple fruit pulp (BRC, KRL, SCT, and SGL) extracts (**b**) prepared with water, ethanol, and methanol.

The IC_50_ values obtained from the ABTS radical scavenging assay for apple peel extracts demonstrated notable variation depending on both the locations and the extraction solvent in Figure 1a. Ascorbic acid (positive control) exhibited the lowest IC_50_ (7.19 ± 0.29 µg/mL). Among the peel extracts, methanolic and ethanolic extracts showed substantially stronger antioxidant activity (lower IC_50_) compared with aqueous extracts. In particular, SGL methanol (IC_50_ = 76.04 ± 0.48 µg/mL), SGL ethanol (78.16 ± 0.51 µg/mL) and SCT ethanol (79.65 ± 0.50 µg/mL) displayed the highest radical scavenging capacities, while BRC methanol (80.40 ± 0.44 µg/mL) showed comparable activity. Conversely, aqueous extracts such as SCT water (114.6 ± 0.59 µg/mL), KRL water (152.1 ± 0.63 µg/mL), and BRC water (172.4 ± 0.70 µg/mL) exhibited markedly weaker activity. Although BRC ethanol extract (127.0 ± 0.62 µg/mL) showed lower activity compared to other ethanolic extracts, overall results indicate that polar organic solvents (methanol/ethanol) are more efficient than water for extracting phenolic and other antioxidant constituents [50,57].

When we looked at the IC_50_ values from the ABTS radical scavenging assay in terms of apple pulp extracts, we saw that the antioxidant activity was very different depending on the solvent used in Figure 1b. Methanolic extracts demonstrated the highest radical scavenging capacity, with BRC methanol (115.0 ± 0.55 µg/mL), KRL methanol (133.0 ± 0.64 µg/mL), and SCT methanol (134.9 ± 0.88 µg/mL) exhibiting significantly lower IC_50_ values in comparison to other samples. Ethanol extracts exhibited moderately reduced yet significant activity, notably KRL ethanol (150.3 ± 0.78 µg/mL) and SCT ethanol (137.0 ± 0.82 µg/mL), whereas aqueous extracts displayed the least antioxidant capacity. The SCT water (184.8 ± 0.85 µg/mL) had the most activity, while the SGL water (723.9 ± 0.84 µg/mL) had the least. While BRC ethanol (178.5 ± 0.74 µg/mL) showed a little less activity than the other ethanolic extracts, the overall trend is clear: organic solvents like methanol and ethanol are better than water at getting phenolic and other antioxidant compounds out of apple pulps. As expected, ascorbic acid, which was used as a positive control, had the most activity (7.19 ± 0. 29 µg/mL).

The IC_50_ values derived from the ABTS radical scavenging assay exhibited significant variations contingent upon both the cultivation site and the specific plant part. In general, methanolic and ethanolic extracts had much stronger antioxidant activity than aqueous extracts. This shows that polar organic solvents are better at getting phenolic and other antioxidant compounds out of apple tissues.

**Figure 2:**
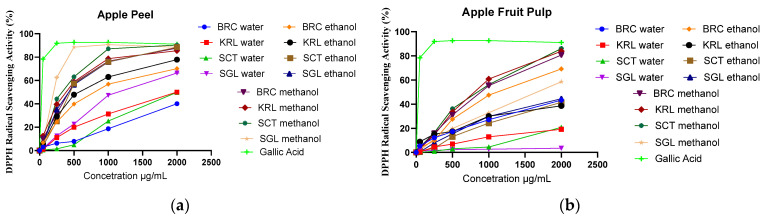
DPPH activity (µg/mL) of apple peel (BRC, KRL, SCT, and SGL) extracts (**a**) and apple fruit pulp (BRC, KRL, SCT, and SGL) extracts (**b**) prepared with water, ethanol, and methanol.

The DPPH radical scavenging assay results revealed notable variations in antioxidant activity among the apple peel extracts depending on the extraction solvent in Figure 2a. Methanolic extracts exhibited the strongest antioxidant capacity, with SGL methanol (185.5 ± 1.7 µg/mL) showing the lowest IC_50_ value, followed by SCT methanol (315.1 ± 1.44 µg/mL), KRL methanol (348.3 ± 1.09 µg/mL) and BRC methanol (409.3 ± 1.27 µg/mL). Ethanolic extracts demonstrated slightly weaker but still considerable activity; in this group, SGL ethanol (406.0 ± 1.27 µg/mL) and SCT ethanol (456.6 ± 1.55 µg/mL) were the most active. In contrast, aqueous extracts displayed the weakest antioxidant potential, as evidenced by their markedly higher IC_50_ values, particularly BRC water (2727 ± 1.38 µg/mL). These findings suggest that methanol and ethanol are more efficient solvents than water for extracting phenolic and other antioxidant compounds from apple peel. Consistent with expectations, gallic acid, used as a positive control, exhibited the highest radical scavenging activity with an IC_50_ value of 1.995 ± 0.43 µg/mL. Overall, the DPPH results corroborate the ABTS assay findings, confirming the superior efficiency of polar organic solvents in recovering antioxidant constituents from apple matrices.

According to the DPPH assay results for apple pulp extracts, significant differences in antioxidant capacity were observed depending on the solvent type and location in Figure 2b. Methanolic extracts exhibited the strongest radical scavenging activity, with SCT methanol (750.0 ± 1.53 µg/mL) and KRL methanol (753.3 ± 1.63 µg/mL) showing the lowest IC_50_ values. Ethanolic extracts showed moderate antioxidant potential, while aqueous extracts presented very weak activity, particularly KRL water (11,598 ± 0.81 µg/mL) and SGL water (47,594,254 ± 0.33 µg/mL). These findings indicate that the extraction efficiency of antioxidant compounds from apple pulp strongly depends on solvent polarity, with methanol being the most effective [50]. Compared with the peel extracts, the fruit pulp exhibited markedly higher IC_50_ values, confirming that phenolic compounds are predominantly accumulated in the outer layers of the fruit.

When the differences between apple peel and pulp and location are evaluated, distinct variations in antioxidant capacity become apparent. In general, the peel exhibited significantly higher phenolic content and, consequently, stronger antioxidant activity than the fruit pulp [51]. This can be attributed to the accumulation of phenolic compounds and flavonoids in the outer cellular layers of the fruit, particularly in the peel tissue [58]. Both ABTS and DPPH assay results consistently showed that peel extracts possessed lower IC_50_ values, indicating greater radical scavenging capacity compared to the corresponding fruit extracts. Moreover, notable differences were also observed among the apple locations. Specifically, the SGL location demonstrated the highest antioxidant activity, yielding the lowest IC_50_ values in both methanolic and ethanolic extracts, while the BRC location exhibited the weakest activity with considerably higher IC_50_ values. These variations may be explained by the differences in phenolic composition, growing conditions, and phytochemical profiles of the apples. Overall, both the solvent type and the location were identified as key factors influencing the antioxidant potential of apple extracts.

#### 3.2.2. Total Phenol Content (TPC) and Total Flavonoid Content (TFC)

**Figure 3:**
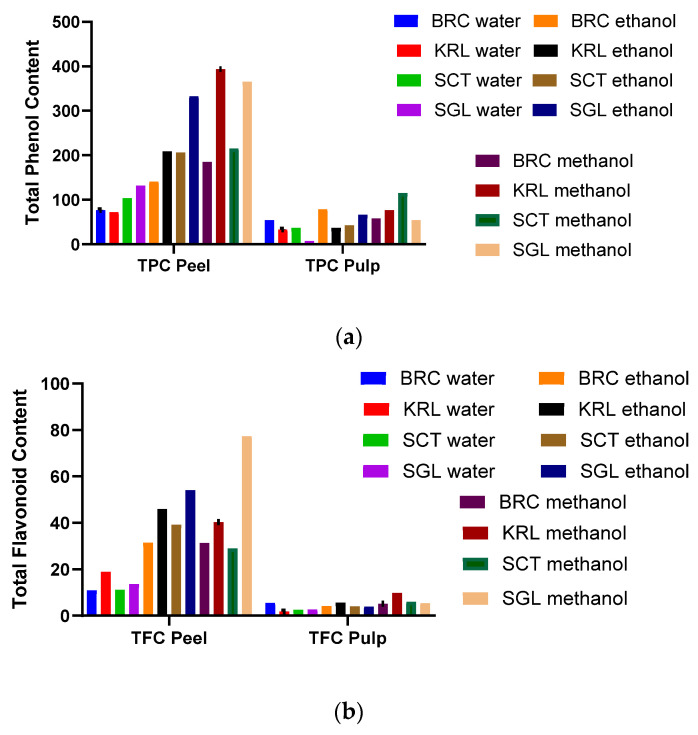
Total Phenol (**a**) and Total Flavonoid Content (**b**) of Apple Peel and Fruit Pulp (BRC, KRL, SCT, and SGL) Prepared With Water, Ethanol, and Methanol.

The total phenolic content (TPC) of apple peel and fruit pulp extracts varied significantly depending on the solvent type and growing location in Figure 3a. In general, peel extracts exhibited considerably higher TPC values than pulp extracts, confirming that phenolic compounds are mainly concentrated in the outer tissues of the fruit. Among the peel samples, methanolic extracts showed the highest TPC levels, particularly KRL methanol (393.4 ± 0.09 mg GAE/g) and SGL methanol (365.4 ± 0.04 mg GAE/g), whereas the lowest content was observed in KRL water extract (72.4 ± 0.07 mg GAE/g).

In contrast, the fruit extracts contained much lower phenolic levels, with SCT methanol (114.8 ± 0.04 mg GAE/g) showing the highest value and SGL water (7.25 ± 0.017 mg GAE/g) the lowest. These results demonstrate that solvent polarity, plant part, and growing location all have a significant influence on phenolic extraction efficiency, and that the peel represents the primary source of phenolic antioxidants in apples. As reported by Schaefer et al. [59], apple phenolic extracts, containing, rutin, caffeic acid, and chlorogenic acid, reduced oxidative DNA damage and intracellular ROS levels.

The phenolic compounds identified in the apple samples, particularly chlorogenic acid, caffeic acid, epicatechin, catechin, quercetin, and rutin, stand out as the primary contributors to antioxidant activity [50,60]. The hydroxyl groups in their chemical structures can neutralize free radicals, thereby reducing oxidative damage and maintaining redox balance [61,62]. Chlorogenic and caffeic acids, belonging to the hydroxycinnamic acid group, are compounds with high radical-scavenging capacity and exhibit strong activity, especially in ABTS and DPPH assays [63,64,65,66]. Flavonoids such as quercetin, rutin, epicatechin, and catechin exert antioxidant effects through both free radical scavenging and metal ion chelation mechanisms [67]. In addition, the compounds identified in apple extracts may exert synergistic effects that enhance total antioxidant capacity, which aligns with the findings of Illam et al. [68] regarding the combined actions of fruit-derived phenolic glycosides against oxidative stress. Therefore, the high accumulation of phenolic compounds in apples is directly associated with the elevated antioxidant activity observed particularly in the peel.

The total flavonoid content (TFC) of apple extracts showed remarkable variations depending on the solvent polarity, plant part, and growing location in Figure 3b. In all samples, peel extracts exhibited markedly higher flavonoid contents than fruit pulps, indicating that flavonoids are primarily concentrated in the epidermal tissues of apples. This is consistent with earlier studies suggesting that flavonoids play a photoprotective role against ultraviolet radiation and oxidative stress, resulting in their accumulation in the peel rather than in the inner tissues [51,54,69].

Among the peel extracts, methanol proved to be the most effective solvent for flavonoid extraction. The highest TFC value was observed in SGL methanol extract (77.36 ± 0.006 mg RE/g), followed by KRL ethanol (45.96 ± 0.02 mg RE/g) and SGL ethanol (54.03 ± 0.02 mg RE/g). These observations are consistent with previous reports indicating that methanol and ethanol possess amphiphilic properties that enhance their ability to dissolve both hydrophilic and hydrophobic compounds, thereby improving the extraction efficiency of phenolics and flavonoids compared to water [50,70]. Conversely, aqueous extracts yielded considerably lower values, with BRC water (10.95 ± 0.007 mg RE/g) showing the weakest flavonoid extraction efficiency, likely due to the limited solubility of many flavonoids in water.

In fruit pulp extracts, the TFC values were significantly lower than those in the peel, further confirming tissue-dependent distribution of flavonoids. The highest pulp TFC was found in KRL methanol (9.82 ± 0.004 mg RE/g), while the lowest was observed in KRL water (1.83 ± 0.001 mg RE/g). Ethanol extracts exhibited moderate activity across all locations, suggesting that solvent composition influences extraction yield but cannot fully overcome tissue-related differences.

Interestingly, regional variation also played a notable role in flavonoid accumulation. The SGL location, characterized by higher altitude and possibly greater sunlight exposure, produced the richest flavonoid profiles in both peel and pulp, supporting the view that environmental stress enhances secondary metabolite biosynthesis. This finding aligns with the report of Ferreyra et al. [69], who highlighted that exposure to environmental stimuli such as light intensity and UV radiation can trigger the activation of flavonoid biosynthetic pathways in plants, leading to increased accumulation of these compounds in tissues exposed to higher stress conditions.

### 3.3. The Anticancer Activity

#### Determination of Cytotoxic Activities of Apple Extracts by MTT Method

**Figure 4:**
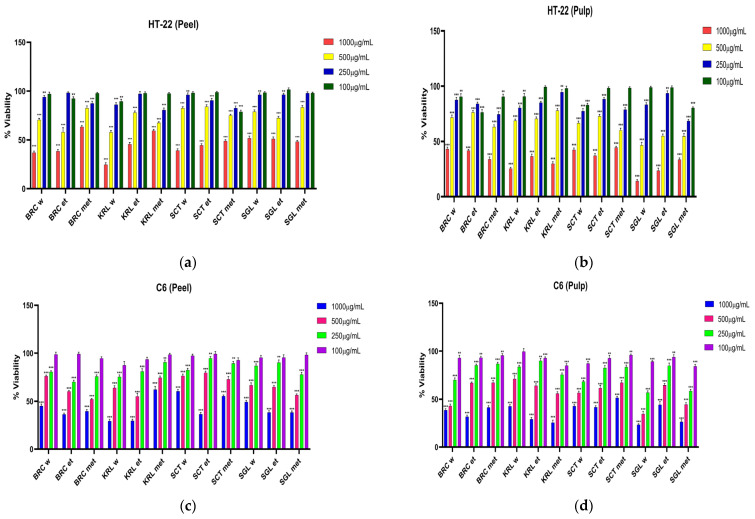
% viability values in HT-22 and C6 cell lines exposed to apple extracts for 24 h: (**a**) HT-22 (Peel); (**b**) HT-22 (Pulp); (**c**) C6 (Peel); (**d**) C6 (Pulp) (*** *p* < 0.001, ** *p* < 0.01).

**Figure 5:**
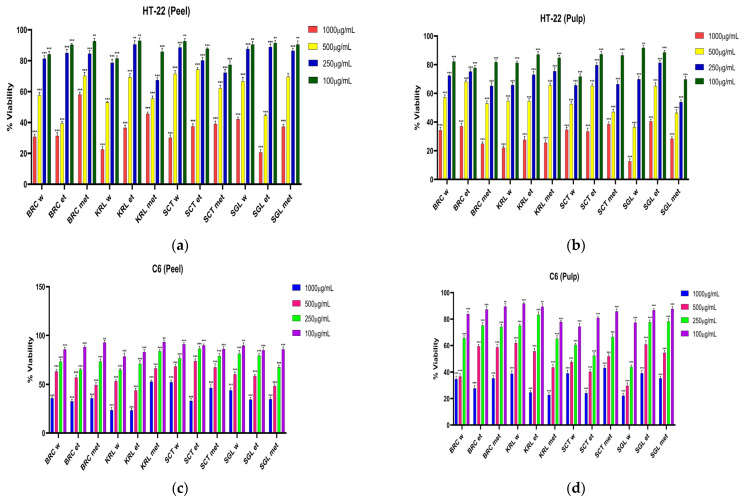
% viability values in HT-22 and C6 celllines exposed to apple extracts for 48 h: (**a**) HT-22 (Peel); (**b**) HT-22 (Pulp); (**c**) C6 (Peel); (**d**) C6 (Pulp) (*** *p* < 0.001, ** *p* < 0.01).

**Figure 6:**
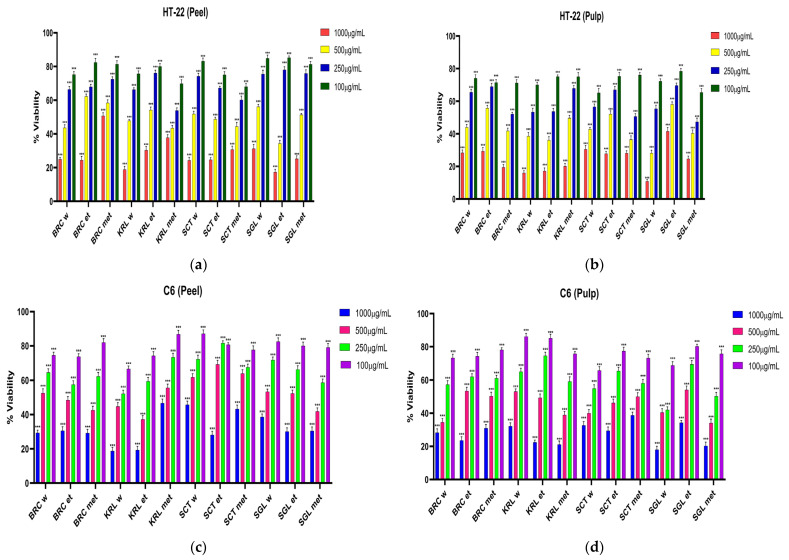
% viability values in HT-22 and C6 cell lines exposed to apple extracts for 72 h: (**a**) HT-22 (Peel); (**b**) HT-22 (Pulp); (**c**) C6 (Peel); (**d**) C6 (Pulp) (*** *p* < 0.001).

In HT-22 and C6 cell lines exposed to apple extracts at doses of 1000 μg/mL, 500 μg/mL, 250 μg/mL, 100 μg/mL, 24, 48 and 72 h IC50 effective doses were determined by the MTT method in Figure 4, Figure 5 and Figure 6 and Table 2, Table 3 and Table 4.

The IC_50_ values obtained after 24 h of incubation revealed significant variations depending on the collection location, solvent type, and plant part. Overall, the fruit pulp extracts exhibited lower IC_50_ values compared to the peel extracts, indicating a higher cytotoxic effect potential. Regarding solvent polarity, methanolic and ethanolic extracts were generally more effective than aqueous ones, which can be attributed to the greater extraction efficiency of polyphenolic and flavonoid compounds in polar organic solvents.

When compared across locations, the SGL samples demonstrated the lowest IC_50_ values in both HT-22 and C6 cell lines, highlighting their strong cytotoxic activity. In particular, the SGL fruit extracts (water and methanol phases, 475.7 and 541.6 µg/mL for HT-22; 338.7 and 387.7 µg/mL for C6, respectively) exhibited the most pronounced inhibitory effects, suggesting that apples from this region may contain higher levels of bioactive compounds. In contrast, the SCT samples exhibited higher IC_50_ values, indicating a weaker cytotoxic effects, while the KRL and BRC samples, although showing values comparable to the other extracts, could be described as displaying moderate levels of activity.

These findings indicate that fruit extracts obtained using polar solvents, especially from the SGL location, possess substantial cytotoxic effect potential. The results further suggest that both the ecological conditions of the growing region and the type of extraction solvent play a crucial role in determining the phenolic profile and biological activity of apple samples.

After 48 h of incubation, the IC_50_ values of apple extracts showed a clear time-dependent decrease compared to the 24 h results, indicating enhanced cytotoxic activity over prolonged exposure. Overall, fruit pulp extracts exhibited lower IC_50_ values than peel extracts, confirming their higher cytotoxic effect potential. Among all samples, the SGL fruit pulp extracts, particularly the methanolic (329.5 µg/mL for HT-22, 609 µg/mL for C6) and aqueous fractions (377.4 µg/mL for HT-22, 242.9 µg/mL for C6), demonstrated the strongest inhibitory effects. In contrast, the SCT samples presented higher IC_50_ values, suggesting weaker activity, whereas the KRL and BRC extracts showed moderate cytotoxic effects. These findings indicate that both extraction solvent polarity and growing location significantly influence the phenolic composition and biological efficacy of apple samples.

After 72 h of incubation, all apple extracts exhibited a further reduction in IC_50_ values compared with the 24 and 48 h results, confirming a time-dependent enhancement of cytotoxicity. Fruit pulp extracts continued to display lower IC_50_ values than peel extracts, indicating a sustained higher cytotoxic effect potential. Among all samples, the SGL fruit pulp extracts, particularly the methanolic and aqueous fractions, retained the strongest inhibitory effects, reaching IC_50_ values below 300 µg/mL (249.6 for water solvent, 239.5 for methanol solvent) in HT-22 and approximately 200 µg/mL (225.9 for water solvent, 269.1 for methanol solvent) in C6 cells. The KRL and BRC samples showed moderate activity, while the SCT extracts maintained comparatively weaker effects. These findings demonstrate that the cytotoxic activity of apple extracts increases with exposure time and is strongly influenced by solvent polarity and growing location.

Tissue-specific distribution of bioactive compounds in apple extracts appears to underlie their distinct biological activities. Analysis of peel and pulp extracts revealed that the peel is particularly rich in epicatechin, rutin, and quercetin, compounds well-known for their potent antioxidant properties. This correlates with the higher antioxidant activity observed in peel extracts. Conversely, the pulp contains higher levels of chlorogenic acid and certain flavonoid combinations, which, despite a lower total antioxidant capacity, may exert stronger anticancer effects through enhanced cytotoxicity and bioavailability in cancer cell lines. These findings underscore the importance of considering tissue-specific phytochemical composition when evaluating the functional potential of fruit extracts, as different compounds contribute differently to antioxidant and anticancer activities.

The anticancer effects of apples are primarily attributed to their phenolic constituents, including phloretin, quercetin and its glycosides, chlorogenic acid, catechin, and epicatechin, as well as triterpenoids predominantly found in the peel. However, these bioactive compounds exhibit low in vivo bioavailability, and clinical evidence remains limited. Since the phytochemical content varies among different parts of the fruit, the consumption of whole apples with their peel and of diverse cultivars may enhance phenolic intake and thereby strengthen the overall anticancer potential [71]. Considering these findings, the high anticancer activity observed in the fruit extracts of the examined apple samples may be attributed to their rich content of phenolic compounds, such as quercetin, chlorogenic acid, catechin, and epicatechin, which possess strong antiproliferative and pro-apoptotic properties. Although no studies have been reported on the effects of apple extracts in HT-22 and C6 cell lines, several investigations have demonstrated the anticancer potential of apples on other cancer cell models such as HeLa, HT-29, and MCF7, indicating the overall cytotoxic and antiproliferative properties of this fruit. In particular, Nezbedova et al. [72] reported that the Granny Smith apple pomace exhibited strong antiproliferative activity against HeLa, HT-29, and MCF7 cancer cell lines, and a significant positive correlation (*p* < 0.05) was observed between its antioxidant capacity and antiproliferative effects, suggesting that its cytotoxicity may be associated with its antioxidant potential. Moreover, although the study indicated that the fruit pulp exhibited slightly higher activity than the peel, the difference between them was not substantial and, in some cases, varied depending on the solvent type and growing location, suggesting that consuming the whole apple with its peel would be more beneficial for maximizing its biological effects.

### 3.4. Molecular Docking and Quantum Chemical Analysis

**Figure 7:**
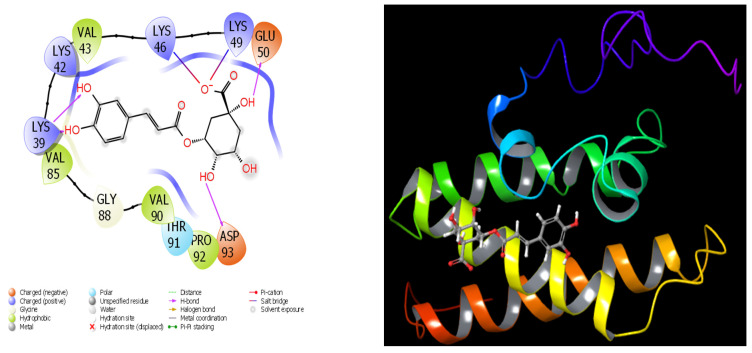
Theoretical interactions of Chlorogenic Acid molecule with PDB ID-2DME.

**Figure 8:**
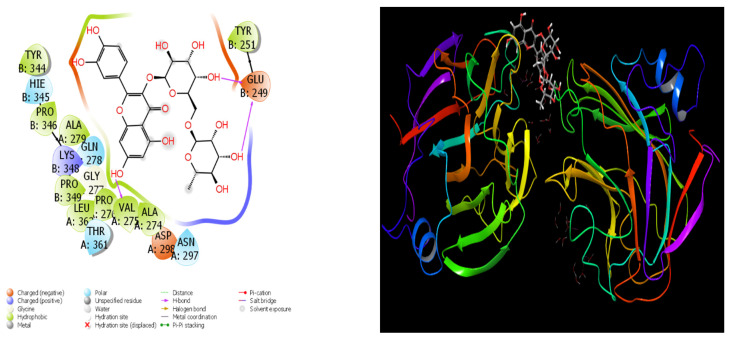
Theoretical interactions of Rutin molecule with PDB ID-2DME.

**Figure 9:**
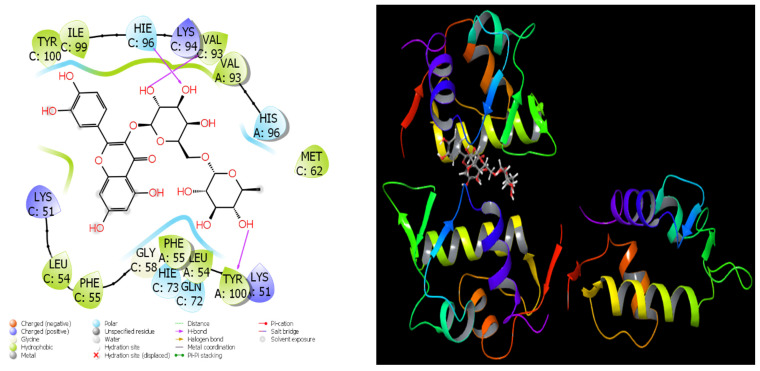
Theoretical interactions of Rutin molecule with PDB ID-1RV1.

Table 5 shows the docking parameters that were used to fully test how well certain phenolic compounds bind to three different proteins (2DME, 6YPE, and 1RV1). These parameters include different energy terms that are used to measure how well the compounds bind to each other and how stable the binding is in Figure 7, Figure 8 and Figure 9.

The docking score shows how well the compound binds to the target protein as a whole. Lower values mean stronger binding. The glide ligand efficiency is the ratio of the binding score to the size of the molecule. This measures how well the molecule binds. Glide Hbond shows how much hydrogen bonds contribute, and the terms vdW (van der Waals) and ecoul (electrostatic energy) show how strong the physical interactions are that happen during binding [73]. The emodel and glide energy show how stable the binding pose and the energy profile are as a whole. Glide is the internal intermolecular tension, and glide posenum is the number of poses that were calculated during pose optimization [74].

Chlorogenic acid (−5.18) has the lowest docking score on 2DME, which means that this compound has the strongest binding affinity for the protein of interest. Chlorogenic acid also helps binding stability by having very negative vdW and electrostatic energy values (−16.74 and −18.4). The glide energy (−52.78) and emodel values (−48.12) are both lower than those of the other compounds, which means that the binding is stable.

Rutin (−4.98) and vitexin (−4.65) are two other compounds that have a strong binding affinity. Rutin is unique because it has a very high negative electrostatic energy of −21.09. Quercetin, epicatechin, and catechin have moderate binding strength, with docking scores between −4.3 and −4.5. Protocatechuic acid seems to be one of the molecules that interacts the least.

Most calculations for 6YPE could not be done for this compound. No valid docking poses were obtained for the other compounds against 6YPE, likely due to incompatibility with the active site geometry under the docking grid parameters used. However, rutin, the only compound that could be calculated, has a very high binding affinity for this protein target, with a docking score of −6.18. The Glide vdW (−31.64), electrostatic energy (−24.79), and especially the Glide energy (−56.43) values calculated for rutin show that the binding is very stable. The Emodel value of −72.87 is very negative, which means that the rutin–protein complex is well optimized.

Rutin (−9.75), catechin (−8.64), and epicatechin (−8.64) all had the strongest binding scores on the 1RV1 protein. Rutin showed the most negative docking score (−9.75) against 1RV1, indicating the strongest binding affinity among all tested compounds. These values indicate that among phenolic compounds, flavonoid structures exhibit a significant affinity for this protein.

Rutin has the lowest values in the docking score and other energy terms by a large margin. Glide energy (−102.89) and emodel (−66.28) show that rutin is very stable. On the other hand, catechin and epicatechin have very negative values for both vdW and electrostatic energy. This means that they support strong hydrogen bonds and interactions between hydrophilic and hydrophobic molecules.

Chlorogenic acid (−6.59) and caffeic acid (−5.60) exhibit moderate binding strengths, while protocatechuic acid (−5.41) and quercetin (−6.02) exhibit relatively weaker binding profiles. Small phenolic acids like caffeic and protocatechuic usually have lower binding scores. This could be because they are small and have a lot of functional groups.

The overall energy parameters show that flavonoid structures (rutin, catechin, epicatechin, and vitexin) make complexes that are stronger and more stable when it comes to both hydrogen bonding and van der Waals interactions [75]. So, flavonoids are the compounds in the table that are most likely to stop target proteins from working.

We looked at the ADME/T analysis results for the chlorogenic acid and rutin molecules with the most activity. We did this by using molecular docking calculations. The ADME/T parameters for chlorogenic acid and rutin elucidate the pharmacokinetic properties of the two molecules. In Table 6, each parameter is explained in academic terms, the two molecules are compared, and reference ranges are set up where they are needed.

Chlorogenic acid has a lower molecular weight than rutin, which has a much higher molecular weight. This indicates that rutin’s bioavailability might be more constrained, given that values exceeding 500 Da are typically linked to inadequate absorption [77]. Rutin has a higher dipole moment, which means that its structure is more polar. When looking at the total surface area (SASA) and the hydrophobic (FOSA) and hydrophilic (FISA, PISA, WPSA) surface components, it is clear that rutin has a much larger surface area, which makes it hard to find a balance between solubility and permeability [78]. The molecular volume of rutin is much bigger, which makes it hard for passive diffusion to get through the cell membrane.

Rutin has more donor and acceptor hydrogen bonds, which usually makes things less permeable. Both molecules have low globularity, which means they do not have spherical shapes. Rutin has a higher polarizability (QPpolrz), which is what you would expect from a large, complex molecule. The logP values for rutin were higher (for example, QPlogPC16, QPlogPoct, and QPlogPw). These values show how the molecule behaves when it moves between different environments. In general, high polarity and high molecular weight can make a molecule less permeable [79].

The solubility parameters (QPlogS and CIQPlogS) for both molecules are very negative, which means that they are not very soluble. However, because the rutin values are lower, it is less soluble than chlorogenic acid. The estimated values for HERG channel inhibition (QPlogHERG) are both negative, which means they are safe. More negative values mean that there is a low risk of cardiotoxicity. Chlorogenic acid has low Caco-2 permeability, but rutin has even lower permeability. Both of these substances are in the poor permeability class when compared to reference values. The brain–blood barrier estimate (QPlogBB) is negative for both molecules, which means that they are not likely to get into the central nervous system [80].

For oral bioavailability, it is bad that MDCK cells do not let rutin through very well. The skin permeation parameter (QPlogKp) is low for both molecules, which means they do not penetrate well. The ionization potential (IP) and electron affinity (EA) values of the two molecules are very similar, which means that they do not react with chemicals in very different ways. Rutin has more metabolizable sites (metab), which means that it may go through more complicated metabolic processes. Both molecules have a low serum albumin binding estimate (QPlogKhsa), which means they do not bind well to plasma proteins.

Chlorogenic acid has a low to moderate level of absorption in the mouth, while rutin has a very low level of absorption. Chlorogenic acid is better than rutin when it comes to estimating percent absorption, but it is still in a range that is considered poor. Rutin has a very high polar surface area (PSA), which is above the reference range. This is in line with its low permeability and low oral bioavailability. Chlorogenic acid is more suitable according to Lipinski’s Rule of Five [81,82], while rutin falls outside the limits on many criteria. Chlorogenic acid also exhibits a simpler molecular profile according to the Rule of Three [83]. Finally, the Jm value is neutral for both molecules, and no significant difference was observed on this parameter.

In general, chlorogenic acid has a limited but better oral bioavailability potential compared to rutin due to its high polarity and moderate solubility. Rutin, on the other hand, has a low absorption profile due to its large, highly polar, and high PSA values, exhibiting significant limitations in both solubility and permeability.

**Figure 10:**
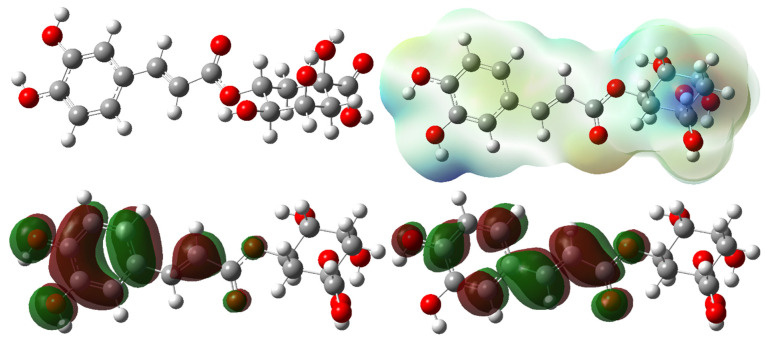
HOMO, LUMO, and ESP of optimized molecular structure of Chlorogenic acid.

**Figure 11:**
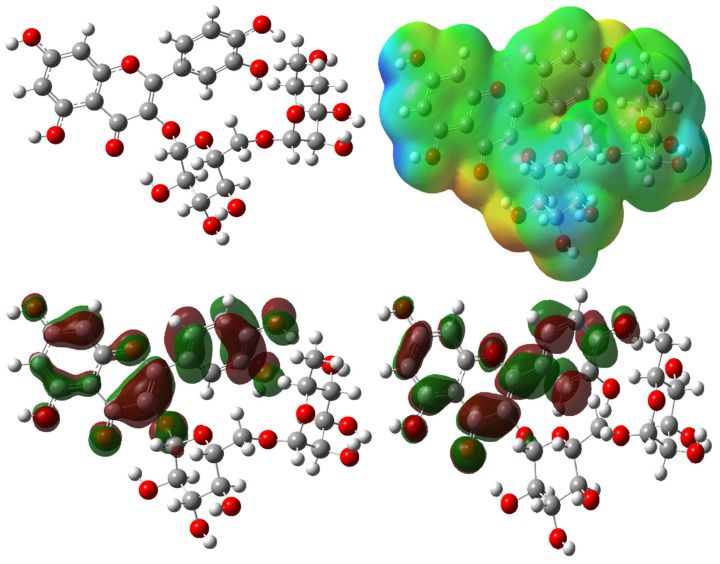
HOMO, LUMO, and ESP of optimized molecular structures of Rutin.

Molecular docking calculations will show the quantum chemical parameters of the two most active molecules, chlorogenic acid and rutin in Figure 10 and Figure 11. Table 7 shows the basic quantum chemical parameters of the molecules that were optimized at different levels (B3LYP, HF, and M06-2X) and with different basis sets (6–31G, 6–31+G, and 6–31+G(d,p)). These parameters give you a lot of information about the molecules’ electronic structures, how they react, how stable they are, and how they move electrons. Below is an explanation of each parameter, followed by an analysis of the general trends among the molecules.

The HOMO energy (E_HOMO_) of a molecule shows how well it can give electrons away. A higher (less negative) HOMO energy means that the molecule is better at giving electrons away. In all methods, the HOMO energy of the Chlorogenic acid molecule is not lower than that of the Rutin molecule [84]. This means that Chlorogenic acid is a better electron donor. As the basis set gets bigger, the HOMO energies go down, which means the molecule is more stable but less likely to react. The LUMO energy (E_LUMO_) shows how likely the molecule is to take in electrons. A lower (more negative) LUMO energy means that the molecule can take in more electrons [85]. The table shows that the LUMO energy of Chlorogenic acid is lower than that of Rutin in all methods. This means that the Chlorogenic acid molecule is more likely to react with nucleophiles.

The ionization potential (I) is the absolute value of the HOMO. It tells you how hard it is for the molecule to lose an electron [84]. There is a big difference between Chlorogenic acid and Rutin, but Rutin usually has a higher I value, which means it is less likely to lose electrons. The electron affinity (A) is the same as the LUMO’s absolute value, and it shows how likely the molecule is to accept electrons [83]. Chlorogenic acid usually has a higher A value, which means it is more likely to accept electrons.

The energy gap ΔE (the difference between the HOMO and LUMO) is one of the most important factors that affect how stable and reactive a molecule is [86]. Lower ΔE values mean that chemicals are more reactive. The ΔE value of Chlorogenic acid is higher than that of Rutin at all levels. This means that Rutin is more reactive and that Chlorogenic acid has a more stable molecular structure. But at the HF level, the gaps are usually bigger, and the HF method gives too much credit to the energy gaps.

Chemical hardness (η) is half of ΔE and shows how resistant a molecule is to changing shape. A system is more stable if its η value is higher, and it is more reactive if its η value is lower [87]. The Chlorogenic acid molecule’s η values are usually higher, which makes the Rutin molecule chemically softer and more reactive.

Electronegativity (χ) is a measure of how likely a molecule is to attract electrons. It is usually equal to half the sum of I and A. The χ values of Chlorogenic acid are higher, which means that Chlorogenic acid has a stronger attraction to electrons and is more electronegative. The chemical potential (μ) is the opposite of electronegativity and tells the system which way the electrons should flow when they are in equilibrium [88]. Negative values are normal and show that the system is stable.

The electrophilic index (ω) tells us how well the molecule can act as an electrophile. The Chlorogenic acid molecule has higher ω values at all levels, which means that Chlorogenic acid is the more common type of molecule in electrophilic reactions [89]. The sign and size of the PA and EA values also show how much the molecules like protons and electrons; higher absolute values of Chlorogenic acid mean that they are better at accepting protons and electrons.

The dipole moment tells us how polar a molecule is and how well it can interact with outside fields. Rutin has a higher dipole moment at every level of calculation. This means that Rutin is more polar and will behave differently when it comes to solubility and binding.

When looking at the total energy values, the energy values of Rutin are more negative in all methods. This means that the Rutin molecule seems to be more stable from a thermodynamic point of view. When the basis set was expanded, the energies moved to more negative values, which made the calculations more stable. Functional comparisons showed that the M06-2X level had wider energy ranges than B3LYP and HF. This means that M06-2X tends to show differences in reactivity and energy more clearly. In general, the Chlorogenic acid molecule stands out because it has a higher electrophilic character, a wider HOMO-LUMO gap, a higher chemical hardness, and a more pronounced electronegativity. The Rutin molecule, on the other hand, has a higher dipole moment, a lower hardness, and a tendency to be more reactive. The findings demonstrate that the electronic disparities between the two isomers are substantial, leading to distinct chemical behaviors regarding reactivity, interactions, and stability.

## 4. Discussion

The present findings demonstrate that the phenolic composition and biological activities of Hünkar apple extracts are strongly influenced by fruit part, geographical origin, and extraction solvent. When compared with previously reported data for other apple cultivars such as Granny Smith, Gala, and Golden varieties, the phenolic profile observed in the current study is consistent with the literature, particularly regarding the predominance of chlorogenic acid, catechin, epicatechin, rutin, and quercetin in apple tissues. Earlier studies have reported that chlorogenic acid is generally the major phenolic compound in apple pulp, whereas flavonols such as quercetin derivatives and rutin are predominantly localized in the peel. Our results confirm this distribution pattern, as peel extracts exhibited higher total phenolic and flavonoid contents and stronger antioxidant capacity, while pulp extracts showed relatively higher chlorogenic acid concentrations and more pronounced cytotoxic activity. The quantitative values obtained in this study fall within or above the ranges reported for many commercial cultivars, suggesting that the Hünkar variety represents a phenol-rich local genotype with significant bioactive potential.

Among the studied regions, the SGL samples consistently demonstrated the strongest antioxidant and anticancer activities. This enhanced activity can be rationalized by the richer and more diverse phenolic profile detected in SGL extracts, particularly in methanolic and ethanolic fractions. Environmental factors such as altitude, sunlight exposure, and abiotic stress are known to stimulate phenylpropanoid metabolism and flavonoid biosynthesis. Therefore, the superior activity of the SGL region may be attributed to ecological conditions that favor secondary metabolite accumulation. Increased UV exposure and environmental stress have been reported to activate key enzymes in the flavonoid biosynthetic pathway, resulting in elevated levels of protective phenolics in fruit tissues. This ecological–biochemical relationship likely underlies the higher antioxidant and cytotoxic responses observed in SGL samples.

An interesting observation in the present study is that anticancer activity was stronger in fruit pulp extracts, whereas antioxidant activity was more pronounced in peel extracts. Although peel contains higher total phenolic and flavonoid levels, the qualitative composition of pulp phenolics appears to favor cytotoxic mechanisms. Chlorogenic acid, which was found at high concentrations in pulp extracts, has been reported to induce apoptosis, modulate oxidative stress pathways, and interfere with cell cycle progression in various cancer models. In contrast, peel-rich flavonols such as rutin and quercetin are particularly effective as radical scavengers due to their multiple hydroxyl groups and conjugated structures. Therefore, the stronger antioxidant activity of peel extracts likely reflects high flavonoid density, while the stronger antiproliferative effect of pulp extracts may be associated with specific phenolic acids and synergistic interactions that more directly modulate intracellular signaling pathways related to apoptosis and proliferation. Additionally, differences in cellular uptake, membrane permeability, and metabolic processing between phenolic subclasses may further contribute to this divergence between antioxidant and anticancer outcomes.

The molecular docking results provide mechanistic support for the experimental anticancer findings. Chlorogenic acid and rutin exhibited favorable binding affinities toward cancer-associated protein targets (2DME, 6YPE, and 1RV1), with rutin showing particularly strong binding scores against 1RV1. These interactions were stabilized by hydrogen bonding, van der Waals forces, and electrostatic contributions, indicating structurally stable ligand–protein complexes. In cancer biology literature, chlorogenic acid has been associated with inhibition of tumor cell proliferation through modulation of PI3K/Akt, MAPK, and mitochondrial apoptotic pathways, while rutin has been reported to suppress angiogenesis, induce apoptosis, and regulate oxidative stress-mediated signaling cascades. The strong docking interactions observed in this study are consistent with these reported molecular mechanisms, suggesting that the identified phenolics may exert anticancer effects by directly interacting with key regulatory proteins involved in tumor progression. Thus, the in silico findings complement the in vitro cytotoxicity data and strengthen the hypothesis that phenolic constituents of Hünkar apples contribute to their observed biological activities at a molecular level.

Overall, the integration of phenolic profiling, antioxidant assays, cytotoxicity evaluation, and molecular docking analysis provides a coherent mechanistic framework explaining the biological potential of Hünkar apple extracts. The results highlight not only the importance of fruit tissue and environmental origin but also the relevance of specific phenolic subclasses in determining antioxidant versus anticancer efficacy.

## 5. Conclusions

This study demonstrates that the biological potential of Hünkar apple extracts is strongly influenced by fruit tissue and geographical origin. Among the investigated regions, the SGL samples consistently showed the most pronounced antioxidant and anticancer activities, indicating that local environmental conditions may significantly enhance phenolic biosynthesis and biological efficacy. This highlights the importance of regional factors in determining the functional value of traditional apple cultivars.

The peel was identified as the richest source of phenolic and flavonoid compounds and exhibited the strongest antioxidant capacity, confirming that the outer fruit tissue represents the primary reservoir of radical-scavenging phytochemicals. In contrast, fruit pulp extracts showed stronger cytotoxic effects in HT-22 and C6 cell lines, suggesting that specific pulp-dominant compounds—particularly chlorogenic acid—may play a more direct role in antiproliferative mechanisms.

Molecular docking results supported the experimental findings, and ADME/T evaluation indicated that chlorogenic acid possesses more favorable drug-likeness properties compared with rutin, particularly in terms of molecular weight, permeability, and Lipinski compliance. Although both compounds demonstrated biological relevance, chlorogenic acid emerged as the more promising candidate for further pharmacological development.

Nevertheless, several limitations should be acknowledged. Cytotoxic activity was assessed solely using the MTT assay in HT-22 and C6 cell lines, which primarily reflects mitochondrial metabolic activity and does not fully represent other cell death pathways. Moreover, in vitro models do not account for bioavailability, metabolism, pharmacokinetics, immune interactions, or systemic toxicity; therefore, the results cannot be directly extrapolated to in vivo conditions. The use of two rodent-derived cell lines also limits generalization to human cancer biology, particularly considering the heterogeneity of brain tumors. Additionally, the marked differences observed among samples collected from different locations underline the influence of environmental factors but also raise concerns regarding extract standardization and reproducibility.

Overall, this integrative experimental and computational approach underscores the therapeutic potential of Hünkar apples as a natural source of bioactive molecules. Future research should include mechanistic apoptosis analyses, additional human cancer cell models, in vivo validation studies, and standardized extract characterization to further clarify the translational potential of chlorogenic acid and related phenolics as complementary anticancer agents.

## Figures and Tables

**Table 1:** The Chemical Composition of apple peel and pulp extracts (BRC, KRL, SCT, and SGL; locations) prepared with water, ethanol, and methanol.

Locations	Solvent	Part	Protokatechuic Acid	Catechin	Chlorogenic Acid	Epicatechin	Caffeic Acid	Syringic Acid	Vitexin	Luteolin-7-Glycoside	Rutin	Quercetin
SCT	Water	peel	256.384	32.128	1612.558	39.259	537.124	128.977	0.383	0.892	ND	ND
	MeOH		ND	825.579	4347.440	2275.822	2.933	205.338	0.921	3.753	377.459	63.993
	EtOH		96.783	1144.866	5305.675	3022.154	10.014	170.067	0.582	3.898	542.194	156.583
	Water	pulp	ND	43.650	4455.379	53.734	16.157	56.821	0.097	ND	ND	ND
	MeOH		ND	252.712	5640.210	288.344	ND	260.257	0.073	1.514	ND	ND
	EtOH		ND	383.934	6753.302	452.357	ND	132.312	0.066	4.394	ND	ND
SGL	Water	peel	2589.102	27.975	2875.782	316.478	57.704	ND	ND	1.642	ND	ND
	MeOH		548.554	371.697	3651.981	1636.208	ND	ND	ND	4.836	545.172	162.450
	EtOH		618.781	389.221	4675.409	1729.013	5.239	ND	ND	6.196	572.923	206.001
	Water	pulp	425.772	ND	249.223	11.698	ND	ND	ND	ND	ND	ND
	MeOH		ND	39.644	430.004	99.030	ND	ND	ND	3.206	ND	ND
	EtOH		7.978	ND	6138.334	0.412	ND	ND	ND	0.043	ND	ND
KRL	Water	peel	232.776	57.884	1159.511	135.397	65.839	ND	ND	ND	ND	ND
	MeOH		181.616	889.080	4852.078	1999.828	6.575	ND	ND	3.344	562.312	75.907
	EtOH		157.656	652.769	6008.434	2706.331	ND	ND	ND	1.981	616.334	132.618
	Water	pulp	391.031	ND	3569.907	3.507	7.529	ND	ND	ND	ND	ND
	MeOH		277.458	61.956	5592.895	89.511	ND	ND	ND	2.045	ND	ND
	EtOH		214.635	118.360	6533.071	176.255	ND	ND	ND	2.592	ND	ND
BRC	Water	peel	112.490	27.391	3474.449	42.893	43.570	ND	ND	ND	ND	ND
	MeOH		28.772	760.236	4867.527	2134.895	ND	ND	ND	ND	328.632	54.566
	EtOH		ND	608.423	5677.433	2403.299	ND	ND	ND	3.452	365.075	62.919
	Water	pulp	69.105	ND	4803.731	2.822	13.218	ND	ND	ND	ND	ND
	MeOH		66.231	235.972	7057.074	317.260	ND	ND	ND	ND	ND	ND
	EtOH		21.720	355.262	7829.797	537.221	ND	ND	ND	2.002	ND	ND

**Table 2:** IC_50_ values of apple extracts in HT-22 and C6 cell lines at 24 h.

	HT-22 IC_50_	C6 IC_50_
BRC(Peel) water	771 ± 26.2 μg/mL	918.6 ± 112.2 μg/mL
BRC(Peel) ethanol	711.9 ± 97.5 μg/mL	638.7 ± 79.6 μg/mL
BRC(Peel) methanol	1628 ± 245.8 μg/mL	626.2 ± 57.3 μg/mL
BRC(Pulp) water	864.6 ± 77.2 μg/mL	520.8 ± 77.3 μg/mL
BRC(Pulp) ethanol	985.8 ± 395.4 μg/mL	687.2 ± 36.5 μg/mL
BRC(Pulp) methanol	648.8 ± 55.8 μg/mL	798.3 ± 28.8 μg/mL
KRL(Peel) water	580.2 ± 41.1 μg/mL	614.5 ± 73.1 μg/mL
KRL(Peel) ethanol	914.6 ± 36.1 μg/mL	579.6 ± 25.8 μg/mL
KRL(Peel) methanol	1330 ± 291.2 μg/mL	1438 ± 224.8 μg/mL
KRL(Pulp) water	638 ± 68.7 μg/mL	839.2 ± 60.6 μg/mL
KRL(Pulp) ethanol	755.7 ± 36.1 μg/mL	658.5 ± 34 μg/mL
KRL(Pulp) methanol	753.1 ± 21.1 μg/mL	529.8 ± 50.2 μg/mL
SCT(Peel) water	855.8 ± 19.5 μg/mL	1515 ± 256.5 μg/mL
SCT(Peel) ethanol	923.6 ± 52.5 μg/mL	816.7 ± 21.7 μg/mL
SCT(Peel) methanol	1283 ± 552.3 μg/mL	1191 ± 125.3 μg/mL
SCT(Pulp) water	839.3 ± 129.8 μg/mL	686.3 ± 54.1 μg/mL
SCT(Pulp) ethanol	780.5 ± 27.3 μg/mL	756.7 ± 44.6 μg/mL
SCT(Pulp) methanol	769 ± 75.9 μg/mL	1006 ± 78.9 μg/mL
SGL(Peel) water	1025 ± 46 μg/mL	940.5 ± 69.9 μg/mL
SGL(Peel) ethanol	983.2 ± 90.6 μg/mL	747.1 ± 39.86 μg/mL
SGL(Peel) methanol	967.5 ± 25.7 μg/mL	656.2 ± 51.8 μg/mL
SGL(Pulp) water	475.7 ± 10.3 μg/mL	338.7 ± 30 μg/mL
SGL(Pulp) ethanol	578.1 ± 31.8 μg/mL	822.5 ± 52.4 μg/mL
SGL(Pulp) methanol	541.6 ± 42.7 μg/mL	387.7 ± 25.1 μg/mL

**Table 3:** IC_50_ values of apple extracts in HT-22 and C6 cell lines at 48 h.

	HT-22 IC_50_	C6 IC_50_
BRC(Peel) water	600.2 ± 64.6 μg/mL	664.8 ± 72.6 μg/mL
BRC(Peel) ethanol	504. 5 ± 75.4 μg/mL	545.6 ± 54.9 μg/mL
BRC(Peel) methanol	1356 ± 145.4 μg/mL	555.8 ± 46.5 μg/mL
BRC(Pulp) water	592.7 ± 51.7 μg/mL	417.4 ± 59.6 μg/mL
BRC(Pulp) ethanol	760.2 ± 196.6 μg/mL	568.7 ± 50.6 μg/mL
BRC(Pulp) methanol	452.8 ± 43.8 μg/mL	626.9 ± 33.4 μg/mL
KRL(Peel) water	504.7 ± 63 μg/mL	434.1 ± 57.8 μg/mL
KRL(Peel) ethanol	756.7 ± 42.5 μg/mL	423.4 ± 32 μg/mL
KRL(Peel) methanol	721 ± 75.9 μg/mL	1054 ± 96.8 μg/mL
KRL(Pulp) water	449.5 ± 57.1 μg/mL	699.7 ± 39.8 μg/mL
KRL(Pulp) ethanol	526.6 ± 32.8 μg/mL	559.1 ± 37.3 μg/mL
KRL(Pulp) methanol	594.1 ± 91.5 μg/mL	381.5 ± 30.1 μg/mL
SCT(Peel) water	708.7 ± 45.1 μg/mL	1108 ± 118.3 μg/mL
SCT(Peel) ethanol	799.9 ± 126.9 μg/mL	742.3 ± 73 μg/mL
SCT(Peel) methanol	716.6 ± 132.9 μg/mL	931.2 ± 128.7 μg/mL
SCT(Pulp) water	498.2 ± 66 μg/mL	473.6 ± 32.5 μg/mL
SCT(Pulp) ethanol	677.7 ± 69.7 μg/mL	323.7 ± 25.7 μg/mL
SCT(Pulp) methanol	525.3 ± 51.4 μg/mL	626.4 ± 66.8 μg/mL
SGL(Peel) water	813 ± 63.2 μg/mL	782 ± 68.9 μg/mL
SGL(Peel) ethanol	496.6 ± 40.9 μg/mL	634.7 ± 63 μg/mL
SGL(Peel) methanol	764.4 ± 59.6 μg/mL	504.7 ± 31.2 μg/mL
SGL(Pulp) water	377.4 ± 10.2 μg/mL	242.9 ± 27.5 μg/mL
SGL(Pulp) ethanol	773.4 ± 59.6 μg/mL	708.1 ± 51.6 μg/mL
SGL(Pulp) methanol	329.5 ± 30.6 μg/mL	609 ± 38.2 μg/mL

**Table 4:** IC50 values of apple extracts in HT-22 and C6 cell lines at 72 h.

	HT-22 IC_50_	C6 IC_50_
BRC(Peel) water	387.7 ± 39.5 μg/mL	453.4 ± 56.5 μg/mL
BRC(Peel) ethanol	525.3 ± 92.3 μg/mL	391 ± 31.9 μg/mL
BRC(Peel) methanol	980.1 ± 119.6 μg/mL	401.5 ± 22 μg/mL
BRC(Pulp) water	396.6 ± 39.7 μg/mL	308.2 ± 29.9 μg/mL
BRC(Pulp) ethanol	494.1 ± 102.1 μg/mL	409.3 ± 66.5 μg/mL
BRC(Pulp) methanol	281.8 ± 27.1 μg/mL	441.5 ± 31.5 μg/mL
KRL(Peel) water	386.9 ± 52.3 μg/mL	269.9 ± 43.8 μg/mL
KRL(Peel) ethanol	546.8 ± 68.1 μg/mL	308.5 ± 24.2 μg/mL
KRL(Peel) methanol	364.2 ± 34.3 μg/mL	770.8± 84.9 μg/mL
KRL(Pulp) water	265.5 ± 26.8 μg/mL	509.8 ± 34.3 μg/mL
KRL(Pulp) ethanol	280.7 ± 14.2 μg/mL	472.2 ± 35.7 μg/mL
KRL(Pulp) methanol	406.5 ± 61.9 μg/mL	323.1 ± 19.5 μg/mL
**SCT(Peel) water**	489.4 ± 44.6 μg/mL	828.3 ± 66.5 μg/mL
**SCT(Peel) ethanol**	418.5 ± 51.1 μg/mL	660.2 ± 124.2 μg/mL
**SCT(Peel) methanol**	357 ± 38.7 μg/mL	829.6 ± 172.3 μg/mL
**SCT(Pulp) water**	310.5 ± 30.3 μg/mL	299.4 ± 27.7 μg/mL
**SCT(Pulp) ethanol**	456.1 ± 59 μg/mL	426.8 ± 31.7 μg/mL
**SCT(Pulp) methanol**	295.8 ± 26.8 μg/mL	474.6 ± 39.6 μg/mL
**SGL(Peel) water**	572.2 ± 45.6 μg/mL	605.6 ± 39.7 μg/mL
**SGL(Peel) ethanol**	399.9 ± 41.6 μg/mL	482.7 ± 38.8 μg/mL
**SGL(Peel) methanol**	495.2 ± 54.8 μg/mL	377.6 ± 23.9 μg/mL
**SGL(Pulp) water**	249.6 ± 22.6 μg/mL	225.9 ± 36 μg/mL
**SGL(Pulp) ethanol**	700.1 ± 68.3 μg/mL	550 ± 42.8 μg/mL
**SGL(Pulp) methanol**	239.5 ± 22.7 μg/mL	269.1 ± 14.7 μg/mL

**Table 5:** Numerical values of the docking parameters of molecules against proteins.

2DME	Docking Score	Glide Ligand Efficiency	Glide Hbond	Glide Evdw	Glide Ecoul	Glide Emodel	Glide Energy	Glide Einternal	Glide Posenum
Caffeic Acid	−4.43	−0.34	−0.24	−7.78	−14.42	−35.74	−22.21	1.61	204
Catechin	−4.5	−0.21	−0.3	−17.91	−12.72	−36.78	−30.63	8.21	14
Chlorogenic Acid	−5.18	−0.21	−0.3	−16.74	−18.4	−52.78	−35.14	3.47	153
Epicatechin	−4.5	−0.21	−0.3	−17.91	−12.72	−36.78	−30.63	8.21	14
Protocatechuic Acid	−4.31	−0.39	0	−12.03	−8.52	−32.33	−20.55	0.57	62
Quercetin	−4.33	−0.2	−0.4	−17.71	−12.3	−38.13	−30.01	3.96	26
Rutin	−4.98	−0.12	−0.31	−23.52	−21.09	−56.07	−44.62	8.92	51
Vitexin	−4.65	−0.15	−0.55	−15.14	−21.03	−47.35	−36.16	6.1	86
6YPE	Docking Score	Glide ligand efficiency	Glide hbond	Glide evdw	Glide ecoul	Glide emodel	Glide energy	Glide einternal	Glide posenum
Caffeic Acid	-	-	-	-	-	-	-	-	-
Catechin	-	-	-	-	-	-	-	-	-
Chlorogenic Acid	-	-	-	-	-	-	-	-	-
Epicatechin	-	-	-	-	-	-	-	-	-
Protocatechuic Acid	-	-	-	-	-	-	-	-	-
Quercetin	-	-	-	-	-	-	-	-	-
Rutin	−6.18	−0.14	−0.32	−31.64	−24.79	−72.87	−56.43	17.29	181
Vitexin	-	-	-	-	-	-	-	-	-
1RV1	Docking Score	Glide ligand efficiency	Glide hbond	Glide evdw	Glide ecoul	Glide emodel	Glide energy	Glide einternal	Glide posenum
Caffeic Acid	−5.60	−0.43	−0.16	−14.06	−11.74	−39.62	−25.80	1.12	92
Catechin	−8.64	−0.41	−0.89	−24.73	−16.61	−59.98	−41.35	3.29	136
Chlorogenic Acid	−6.59	−0.26	−0.14	−31.61	−9.81	−56.68	−41.43	9.67	186
Epicatechin	−8.64	−0.41	−0.89	−24.73	−16.61	−59.98	−41.35	3.29	136
Protocatechuic Acid	−5.41	−0.49	0.00	−11.10	−10.08	−31.40	−21.18	0.32	31
Quercetin	−6.02	−0.27	−0.01	−30.36	−6.43	−49.85	−36.79	0.82	7
Rutin	−9.75	−0.23	0.00	−42.98	−25.12	−102.89	−68.11	8.30	148
Vitexin	−7.72	−0.25	−0.61	−34.61	−15.20	−69.66	−49.81	5.75	142

**Table 6:** ADME properties of the molecules.

	Chlorogenic Acid	Rutin	Referance Range
mol_MW	354	611	130–725
dipole (D)	8.0	10.2	1.0–12.5
SASA	593	743	300–1000
FOSA	120	160	0–750
FISA	330	425	7–330
PISA	143	158	0–450
WPSA	0	0	0–175
volume (A^3^)	1033	1510	500–2000
donorHB	6	9	0–6
accptHB	9.7	20.6	2.0–20.0
glob (Sphere = 1)	0.8	0.9	0.75–0.95
QPpolrz (A^3^)	30.1	46.1	13.0–70.0
QPlogPC16	12.5	18.2	4.0–18.0
QPlogPoct	25.1	41.7	8.0–35.0
QPlogPw	20.4	35.6	4.0–45.0
QPlogPo/w	−0.3	−2.6	−2.0–6.5
QPlogS	−2.3	−1.8	−6.5–0.5
CIQPlogS	−2.9	−4.2	−6.5–0.5
QPlogHERG	−3.1	−4.6	*
QPPCaco (nm/s)	2	1	**
QPlogBB	−3.2	−4.2	−3.0–1.2
QPPMDCK (nm/s)	1	0	**
QPlogKp	−6.1	−7.4	Kp in cm/h
IP (ev)	9.3	9.1	7.9–10.5
EA (eV)	0.9	0.6	−0.9–1.7
#metab	5	10	1–8
QPlogKhsa	−0.9	−1.2	−1.5–1.5
Human Oral Absorption	1	1	-
Percent Human Oral Absorpt.	17	0	***
PSA	182	271	7–200
RuleOfFive	1	3	Maximum is 4
RuleOfThree	1	2	Maximum is 3
Jm	0.0	0.0	-

* concern below −5, ** <25 is poor and >500 is great, *** <25% is poor and >80% is high [76].

**Table 7:** The calculated quantum chemical parameters of molecules.

	E_HOMO_	E_LUMO_	I	A	ΔE	η	μ	χ	PA	ω	ε	Dipol	Energy
**B3LYP/6**–**31g LEVEL**													
**1**	−6.0627	−2.0479	6.0627	2.0479	4.0148	2.0074	0.4982	4.0553	−4.0553	4.0963	0.2441	6.620	−35,289.8829
**2**	−5.6538	−1.8830	5.6538	1.8830	3.7707	1.8854	0.5304	3.7684	−3.7684	3.7661	0.2655	8.331	−61,205.4629
**B3LYP/6**–**31++g LEVEL**													
**1**	−6.4149	−2.4221	6.4149	2.4221	3.9928	1.9964	0.5009	4.4185	−4.4185	4.8896	0.2045	6.598	−35,291.7800
**2**	−6.0464	−2.2624	6.0464	2.2624	3.7840	1.8920	0.5285	4.1544	−4.1544	4.5610	0.2193	8.853	−61,208.5572
**B3LYP/6**–**31++g(d,p) LEVEL**													
**1**	−6.2276	−2.1699	6.2276	2.1699	4.0578	2.0289	0.4929	4.1987	−4.1987	4.3446	0.2302	5.718	−35,303.5994
**2**	−5.9833	−2.0664	5.9833	2.0664	3.9168	1.9584	0.5106	4.0249	−4.0249	4.1359	0.2418	6.848	−61,228.9196
**HF/6**–**31g LEVEL**													
**1**	−8.4895	1.5443	8.4895	−1.5443	10.0337	5.0169	0.1993	3.4726	−3.4726	1.2018	0.8321	6.218	−35,083.4992
**2**	−7.9833	2.1816	7.9833	−2.1816	10.1649	5.0824	0.1968	2.9009	−2.9009	0.8279	1.2079	9.773	−60,849.1527
**HF/6**–**31++g LEVEL**													
**1**	−8.6530	0.7878	8.6530	−0.7878	9.4408	4.7204	0.2118	3.9326	−3.9326	1.6382	0.6104	6.168	−35,084.7228
**2**	−8.1349	0.6272	8.1349	−0.6272	8.7621	4.3811	0.2283	3.7538	−3.7538	1.6082	0.6218	8.464	−60,851.3031
**HF/6**–**31++g(d,p) LEVEL**													
**1**	−8.3540	0.8515	8.3540	−0.8515	9.2054	4.6027	0.2173	3.7513	−3.7513	1.5287	0.6542	5.272	−35,101.1076
**2**	−8.1970	0.6498	8.1970	−0.6498	8.8468	4.4234	0.2261	3.7736	−3.7736	1.6096	0.6213	7.121	−60,879.0950
**M062X/6**–**31g LEVEL**													
**1**	−7.3890	−1.1489	7.3890	1.1489	6.2402	3.1201	0.3205	4.2690	−4.2690	2.9204	0.3424	6.527	−35,276.3589
**2**	−6.7507	−0.7271	6.7507	0.7271	6.0236	3.0118	0.3320	3.7389	−3.7389	2.3208	0.4309	7.780	−61,182.6816
**M062X/6**–**31++g LEVEL**													
**1**	−7.6549	−1.4721	7.6549	1.4721	6.1827	3.0914	0.3235	4.5635	−4.5635	3.3684	0.2969	6.497	−35,277.9242
**2**	−7.0500	−1.0727	7.0500	1.0727	5.9773	2.9887	0.3346	4.0613	−4.0613	2.7595	0.3624	7.884	−61,185.3956
**M062X/6**–**31++g(d,p) LEVEL**													
**1**	−7.4533	−1.2049	7.4533	1.2049	6.2483	3.1242	0.3201	4.3291	−4.3291	2.9994	0.3334	5.531	−35,289.5470
**2**	−7.0685	−0.7415	7.0685	0.7415	6.3270	3.1635	0.3161	3.9050	−3.9050	2.4102	0.4149	9.125	−61,205.1874

1: Chlorogenic acid, 2: Rutin.

## Data Availability

The raw data supporting the conclusions of this article will be made available by the authors on request.
